# High Case-Fatality Rate for Human Anthrax, Northern Ghana, 2005–2016

**DOI:** 10.3201/eid2704.204496

**Published:** 2021-04

**Authors:** Jason K. Blackburn, Ernest Kenu, Franklin Asiedu-Bekoe, Badu Sarkodie, Ian T. Kracalik, William A. Bower, Robyn A. Stoddard, Rita M. Traxler

**Affiliations:** University of Florida, Gainesville, Florida, USA (J.K. Blackburn, I.T. Kracalik);; University of Ghana, Accra, Ghana (E. Kenu);; Ghana Health Service, Accra (F. Asiedu-Bekoe, B. Sarkodie);; Centers for Disease Control and Prevention, Atlanta, Georgia, USA (W.A. Bower, R.A. Stoddard, R.M. Traxler)

**Keywords:** Africa, Bacillus anthracis, bacteria, case-fatality rate, cutaneous anthrax, enteric infections, food safety, gastrointestinal anthrax, Ghana, human anthrax, livestock, livestock vaccination, mortality, surveillance

## Abstract

The human cutaneous anthrax case-fatality rate is ≈1% when treated, 5%–20% when untreated. We report high case-fatality rates (median 35.0%; 95% CI 21.1%–66.7%) during 2005–2016 linked to livestock handling in northern Ghana, where veterinary resources are limited. Livestock vaccination and access to human treatment should be evaluated.

Despite being one of the earliest diseases described, anthrax, caused by infection with the bacterium *Bacillus anthracis*, remains a global public health concern, especially in resource-limited, rural agricultural areas, including West Africa (*1*). Cutaneous anthrax, the most common human form, is readily treatable with antimicrobials and has a case-fatality rate (CFR; annual anthrax deaths/annual anthrax cases) ≈1%. Left untreated, CFR increases to 5%–20% for cutaneous anthrax and 25%–60% for gastrointestinal anthrax (*1*,*2*). However, Ghana has a history of human anthrax associated with high CFRs. One study reported that nearly 1,000 persons died from anthrax in Ghana during 1980–2000 (*3*). Most cases occurred in northern Ghana and were attributed to spillover from infected livestock. A 2017 study modeling the geographic distribution of anthrax risk in Ghana corroborated those findings and identified Northern, Savannah, Upper West, North East, and Upper East regions (using the current nomenclature for regions) as the areas of greatest risk for anthrax persistence in livestock and associated risk to humans (*4*). A 2000 study (*3*) focusing on Tamale, a major livestock production and trading center in northern Ghana, identified behaviors that increased risk for acquiring anthrax infection. These behaviors included neglecting livestock vaccination despite local vaccine production and availability, slaughtering, butchering, and distributing the meat from livestock that were sick or had died from anthrax, and believing that cooking the meat with specific herbs makes it safe to consume. In addition, the use of untested herbal or holistic treatments to cure anthrax has increased CFR; use of plants to treat anthrax has also been described in Nigeria (*5*).

Whereas human and livestock anthrax outbreaks are reported nearly annually in Ghana (*3*,*4*), most available studies are dated or limited to the distribution of human cases within specific communities or over short time periods (*6*). Here we describe human anthrax cases and resulting deaths reported across northern Ghana during 2005–2016.

## The Study

We obtained data for the study through a One Health collaboration of the Ghanaian Ministries of Statistics, Health and Veterinary Services, and Agriculture, as part of anthrax surveillance capacity building aimed at integrating livestock and human reporting (*4*). Data included field reports not previously aggregated in national reports, reviews of national reports filed with the appropriate ministries, and outbreak investigations captured in gray literature or peer-reviewed articles. We used livestock data published elsewhere (*4*,*7*). Ethics approval was received by the Noguchi Memorial Institute for Medical Research in Accra.

Human anthrax cases were reported by district in Upper East region and a single district in Northern region. Data from Upper East were limited to aggregated annual counts per district for 2005–2015; limited line list data were available for 2016. Case-patient age and sex were available in Upper East for a subset of years, 2008–2014; other regions included human anthrax fatalities by month and year during 2005–2016. Deaths were aggregated by month and by year for comparison to livestock reports. No information was available on the form of infection (e.g., cutaneous, gastrointestinal).

We calculated CFRs and exact binomial CIs using the epitools package in the R software package (The R Project for Statistical Computing, https://www.r-project.org). We calculated median CFRs with bias-corrected and accelerated CIs with 10,000 replicates using the boot package in R. Because Upper East data were more complete, we calculated CFRs by district and year.

During 2005–2016, a total of 38 human deaths from anthrax were reported in Ghana, 30 from Upper East and 8 from Northern regions. For 8 cases with month of occurrence documented, deaths were reported in March, April, June, and December. Four deaths occurred during March and April, and in the Upper East, corresponding to seasonal peaks and geographic concentrations of livestock anthrax (*4*,*7*).

District-level data reported 30 (36.1%) deaths from 83 human anthrax cases in Upper East, including 1 district, West Mamprusi (now part of North East; Figure). During 2005–2016, Bawku West reported outbreaks in 4 years, Talensi Nabdam in 3 years, and other districts in 1 year. Cases of human anthrax peaked in 2006, 2008, and 2014 (Table). One study reported 43 livestock anthrax outbreaks in Northern region during 2005–2012; in 6 (14.0%) of those outbreaks, human cases were reported (*8*). From the 28 cases of cutaneous human anthrax in those 6 outbreaks, 6 (22.2%) persons died. However, neither case counts nor mortality rates could be directly associated with other reported livestock outbreaks. This disconnection of data on human anthrax cases from livestock outbreaks has been documented elsewhere (*9*). In data from Upper East with information on the age and sex of case-patients, 75.6% (31/41) of patients were men, consistent with rates in a previous report (*6*). Of the 31 men, 48.4% (15/31) died compared with 40.0% (4/10) of women. For all cases with age data, median age was 38 years (range 7–81 years) for men and 39.5 years (range 4–61 years) for women.

**Figure:**
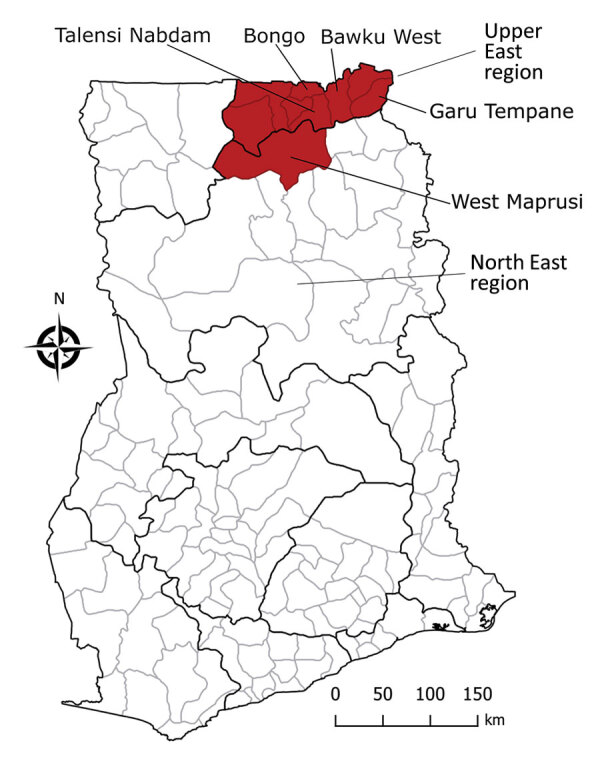
Districts in northern Ghana reporting human anthrax case and mortality data during 2005–2016.

**Table:** Annual human mortality by district for Upper East region and a single district from what is now North East region (formerly part of Northern region), Ghana, 2006–2016

Region and district	Year	Cases	Deaths	Case-fatality rate (95% CI)
Upper East				
Bawku West	2006	27	6	22.2 (8.6–42.3)
	2012	3	2	66.7 (9.4–99.2)
	2013	7	6	85.7 (42.1–99.6)
	2014	1	1	100.0 (2.5–100.0)
Garu-Tempane	2008	10	3	30.0 (6.7–65.2)
Talensi Nabdam	2008	6	1	16.7 (0.4–64.1)
	2009	9	2	22.2 (2.8–60.0)
	2014	10	6	60.0 (26.2–87.8)
Bongo	2012	5	2	40.0 (5.3–85.3)
North East*				
West Mamprusi	2007	5	1	20.0 (0.5–71.6)

*Current region for this district.

## Conclusions

We found CFRs from recurrent anthrax outbreaks associated with human deaths in northern Ghana exceeded expected global CFRs for treated and untreated cutaneous anthrax and were likely untreated gastrointestinal anthrax. Evidence of the consumption of anthrax-contaminated meat in the region suggests gastrointestinal anthrax is probably underrecognized in Ghana (*1*,*2*,*10*). The very high death rates may indicate that gastrointestinal anthrax is frequent in Ghana (*1*), which is supported by findings regarding beliefs in the same area about what meat is safe to consume (*3*). A study in Tamale, Ghana, found that livestock dying from anthrax are considered a ready source of meat for local community members, potentially leading to high human case numbers from relatively few animals (*3*). More education about the risks of consuming meat from animals that die suddenly is needed to counteract this belief.

The form of anthrax in cases identified here was undocumented. However, CFRs for human anthrax in Ghana were higher than for outbreaks in Thailand (CFR = 4%) and Zambia (CFR = 19%) involving gastrointestinal anthrax (*10*,*11*). High anthrax CFRs highlight 2 challenges. First, access to treatment may be limited, requiring long-distance travel in some settings (*11*). Second, anthrax underreporting is common, particularly for gastrointestinal anthrax because symptoms are atypical. There is insufficient awareness among healthcare providers and limited diagnostic capacity in rural endemic areas; anthrax should be included in differential diagnoses.

Although not captured in the data here, 2 deaths in March 2016 in Ghana were reported as suspected anthrax in the human health reporting system but could not be laboratory confirmed (data not shown). Both were reported as anthrax based on clinical signs and an epidemiologic link to livestock clinically di-agnosed by the veterinary reporting system. This in-cident, coupled with findings from our analyses, il-lustrates the need for joint evaluation of records and uniformity in case definitions between human and animal reporting systems. 

Early treatment is crucial for recovery from anthrax; prophylactic antimicrobial drugs are recommended for persons with known or suspected exposure to anthrax-contaminated meat or livestock. Livestock vaccination remains the most effective control method for reducing anthrax burden in livestock and humans (*12*,*13*); however, minimal vaccination uptake persists because of beliefs about its effectiveness and limited veterinary services and because underreporting associated with diagnostic capacity hinders vaccination campaigns. Recent models prioritized geographic areas for prevention and control (*1*,*4*).

Our findings highlight the need for education about the risks of consuming meat from sick or dead animals and the benefits of livestock vaccination, increased healthcare provider awareness, and evaluation of accessibility to anthrax treatment. These findings support ongoing efforts in Ghana to coordinate human and livestock anthrax reporting and to improve and expand diagnostic capacity.

## References

[1] Carlson CJ, Kracalik IT, Ross N, Alexander KA, Hugh-Jones ME, Fegan M, et al. The global distribution of *Bacillus anthracis* and associated anthrax risk to humans, livestock and wildlife. Nat Microbiol. 2019;4:1337–43. 10.1038/s41564-019-0435-4 31086311

[2] Doganay M, Metan G. Human anthrax in Turkey from 1990 to 2007. Vector Borne Zoonotic Dis. 2009;9:131–40. 10.1089/vbz.2008.0032 18945187

[3] Opare C, Nsiire A, Awumbilla B, Akanmori BD. Human behavioural factors implicated in outbreaks of human anthrax in the Tamale municipality of northern Ghana. Acta Trop. 2000;76:49–52. 10.1016/S0001-706X(00)00089-9 10913766

[4] Kracalik IT, Kenu E, Ayamdooh EN, Allegye-Cudjoe E, Polkuu PN, Frimpong JA, et al. Modeling the environmental suitability of anthrax in Ghana and estimating populations at risk: Implications for vaccination and control. PLoS Negl Trop Dis. 2017;11:e0005885. 10.1371/journal.pntd.0005885 29028799 PMC5656412

[5] Alawa JP, Jokthan GE, Akut K. Ethnoveterinary medical practice for ruminants in the subhumid zone of northern Nigeria. Prev Vet Med. 2002;54:79–90. 10.1016/S0167-5877(01)00273-2 12062521

[6] Awoonor-Williams JK, Apanga PA, Anyawie M, Abachie T, Boidoitsiah S, Opare JL, et al. Anthrax outbreak investigation among humans and animals in northern Ghana: case report. Int J Trop Dis Health. 2015;12:1–11. 10.9734/IJTDH/2016/22359

[7] Nsoh AE, Kenu E, Forson EK, Afari E, Sackey S, Nyarko KM, et al. Mapping as a tool for predicting the risk of anthrax outbreaks in Northern Region of Ghana. Pan Afr Med J. 2016;25(Suppl 1):14. 10.11604/pamj.supp.2016.25.1.6205 28149439 PMC5257015

[8] Ayamdooh EN. Mapping as a tool for predicting the risk of anthrax outbreaks in Northern Region of Ghana [thesis]. Accra (Ghana): University of Ghana; 2015.

[9] Sirisanthana T, Brown AE. Anthrax of the gastrointestinal tract. Emerg Infect Dis. 2002;8:649–51. 10.3201/eid0807.020062 12095428 PMC2730335

[10] Siamudaala VM, Bwalya JM, Munang’andu HM, Sinyangwe PG, Banda F, Mweene AS, et al. Ecology and epidemiology of anthrax in cattle and humans in Zambia. Jpn J Vet Res. 2006;54:15–23. 16786974

[11] Kracalik I, Malania L, Tsertsvadze N, Manvelyan J, Bakanidze L, Imnadze P, et al. Human cutaneous anthrax, Georgia 2010-2012. Emerg Infect Dis. 2014;20:261–4. 10.3201/eid2002.130522 24447721 PMC3901487

[12] Kracalik I, Abdullayev R, Asadov K, Ismayilova R, Baghirova M, Ustun N, et al. Changing patterns of human anthrax in Azerbaijan during the post-Soviet and preemptive livestock vaccination eras. PLoS Negl Trop Dis. 2014;8:e2985. 10.1371/journal.pntd.0002985 25032701 PMC4102439

[13] Kracalik I, Malania L, Broladze M, Navdarashvili A, Imnadze P, Ryan SJ, et al. Changing livestock vaccination policy alters the epidemiology of human anthrax, Georgia, 2000-2013. Vaccine. 2017;35:6283–9. 10.1016/j.vaccine.2017.09.081 28988866

